# Expression Levels of the Uridine-Cytidine Kinase Like-1 Protein As a Novel Prognostic Factor for Hepatitis C Virus-Associated Hepatocellular Carcinomas

**Published:** 2017

**Authors:** A. Buivydiene, V. Liakina, J. Valantinas, J. Norkuniene, E. Mockiene, S. Jokubauskiene, R. Smaliukiene, L. Jancoriene, L. Kovalevska, E. Kashuba

**Affiliations:** Vilnius University, Clinic of Gastroenterology, Nephrourology and Surgery, Centre of Hepatology, Gastroenterology and Dietetics, Vilnius, Lithuania; Vilnius University, Center of Hepatology, Gastroenterology and Dietetics, Vilnius, Lithuania; Vilnius Gediminas Technical University, Department of Biomechanics, Vilnius, Lithuania; Vilnius Gediminas Technical University, Department of Mathematical Statistics, Vilnius, Lithuania; Vilnius College of Higher Education, Vilnius, Lithuania; Centre of Radiology and Nuclear Medicine, Vilnius, Lithuania; Vilnius University, Faculty of Medicine, Vilnius, Lithuania; Vilnius University, Department of Pathology, Forensic Medicine and Pharmacology, Vilnius, Lithuania; National Center of Pathology, Vilnius, Lithuania; Vilnius University, Clinic of Infectious, Chest Diseases, Dermatovenerology and Allergy, Center of Infectious Diseases, Vilnius, Lithuania; R.E. Kavetsky Institute of Experimental Pathology, Oncology and Radiobiology, Kyiv, Ukraine; Department of Microbiology, Tumor and Cell Biology, Karolinska Institutet, Stockholm, Sweden

**Keywords:** hepatitis C virus (HCV), hepatocellular carcinoma (HCC), recurrence of hepatocellular carcinoma, UCKL-1, MRPS18-2, prognostic factors

## Abstract

The expression levels of the two novel oncoproteins uridine-cytidine kinase like-1 (UCKL-1) and mitochondrial ribosomal protein S18-2 (MRPS18-2) were assessed in samples of hepatitis C virus (HCV)-associated hepatocellular carcinoma (HCC) using immunohistochemistry. Tissue microarray (TMA) paraffin blocks were prepared from 42 HCC tumor samples with the corresponding peri-tumor tissues and from 11 tissues of a liver with HCV-induced cirrhosis. We found that the UCKL-1 signal in the liver tissues of the peri-tumor zone in the HCC samples was stronger than that in cirrhosis (50 ± 49.44 vs. 24.27 ± 14.53; *p *= 0.014). The MRPS18-2 expression was weak, and there was no differences between the groups (*p *= 0.26). Noteworthy, the UCKL-1 protein was expressed at higher levels in peri-tumor tissues in the cases of HCC recurrence; this was confirmed for 27 older patients (63.78 ± 9.22 vs. 53.53 ± 4.07 years, *p* < 0.001), in parallel with enhanced UCKL-1 staining in former HCC nodules (62.69 ± 50.4 vs. 26.0 ± 30.19, *p *= 0.006) and microvascular invasion (*p *= 0.02). A multivariate analysis of prognostic factors for HCC recurrence showed that the best predictive factors for these conditions were UCKL-1 expression in tumor, vascular invasion, and HCC treatment modality, other than liver transplantation (odds ratios: 1.029, 18.143 and 11.984, *R*^2^ = 0.633, *p *= 0.002). In conclusion, the high UCKL-1 expression might be a prognostic factor for HCC relapse, in combination with age and microvascular invasion. MRPS18-2 protein expression has no prognostic significance in the cases of HCV-associated HCC.

## INTRODUCTION

Liver cancer (predominantly HCC) is the second-most deadly cancer for men worldwide [1]. HCC incidence in Nordic countries, including Lithuania, reaches up to 10/100,000 inhabitants [2]. In developed countries, one factor that is responsible for the increased HCC incidence is HCV [3]. In Lithuania, anti-HCV prevalence in adults stands at about 2.78% [4]. HCV-induced HCC development is a multi-step process that may last 20–40 years and involves chronic hepatic inflamma tion, progressive liver fibrosis, initiation of neoplastic clones, and tumor progression in a carcinogenic tissue microenvironment [5]. Noteworthy, eradication of HCV reduces, but does not eliminate, the risk of HCC development, especially when advanced hepatic fibrosis has already originated [6, 7]. It becomes difficult then and hardly manageable to follow patients at early-stage asymptomatic HCV-induced cirrhosis. Thus, it was discovered in a population-based study that less than 20% of patients with cirrhosis who had developed HCC were subject to regular monitoring [8]. Therefore, prognostic markers are now being actively developed to stratify HCV patients into clearly defined risk groups. Successful employ of these predictors in clinical practise could improve the clinical management of these patients [9]. Additionally, even with a successful HCC treatment by liver transplantation, liver resection, or radiofrequency-induced thermotherapy (RFITT), high risk of HCC recurrence persists [7, 8]. The recurrence rate of HCC is estimated at 70% after 5 years of liver resection [10]. The validated prediction markers of recurrence are the tumor size, multifocality, macroscopic and microscopic vascular invasion, as well as poor differentiation [11].

It is widely accepted that malignant transformation of liver cells is stimulated by various factors. However, studies on the mechanisms of HCV-induced cell transformation are inhibited by the lack of animal and cell models. Obviously, better understanding of molecular mechanisms would help us identify new diagnostic and/or prognostic markers, most importantly, for the early detection of HCC [11]. This would allow us to develop better approaches to clinical treatment.

Usually, the molecular mechanisms that are responsible for virus-induced cell transformation include the inactivation of the two tumor suppressor protein pathways: i.e., the p53 (TP53) and retinoblastoma (RB) pathways [12, 13]. There is little doubt that other proteins can play an important role in cell transformation: for example, the putative human enzyme UCKL-1 that is involved in cellular nucleotide metabolism [14, 15] and the new oncoprotein MRPS18-2 that can bind RB [13, 16, 17]. There are no data on the expression of both of these proteins in HCC, and we asked ourselves the question of whether the UCKL-1 and MRPS18-2 expressions in HCC tissues could be used as prognostic markers for the course of the disease in HCV-bearing patients.

## EXPERIMENTAL

**Patient samples**

The retrospective cohort study was conducted at Vilnius University Hospital Santariskiu Klinikos, Vilnius, Lithuania, according to the guidelines of the Helsinki Declaration. The study was approved by the Vilnius Regional Biomedical Research Ethics Committee (158200-13-698-224, from 2013-11-12). The HCC tumor and the corresponding peri-tumor (normal) liver tissue samples were collected from 53 patients who had undergone liver transplantation, liver resection, or RFITT for a complicated chronic HCV infection. Tissue sections from 42 HCV positive cirrhotic patients with HCC and 11 samples from transplanted HCV cirrhotic patients without HCC, as a control group, were analyzed. All the specimens are preserved at the National Lithuanian Center of Pathology. A histological activity index (HAI) was scored, according to K. Ishak et al., and liver fibrosis was assessed, according to METAVIR [18-20]. In HCC cases, tumor differentiation and microvascular invasion were evaluated.

**Analysis of UCKL-1 and MRPS18-2 expression in HCC and liver tissue specimens**

Tissue samples after surgery, hepatic resection, and/ or liver transplantation were fixed in a buffered 10% formalin solution. Expression of the UCKL-1 and MRPS18-2 proteins was performed by immunohistochemistry (IHC) on tissue microarrays (TMAs) constructed of paraffin-embedded tissues, selected by the pathologist. Cores one millimeter in diameter were punched from the selected areas. Paraffin sections of the TMAs were cut (2 μm thick), dewaxed, deparaffinized, and rehydrated. Epitopes were heat-activated in a EnVision FLEX target retrieval solution for 20 min, while the pH of the buffer was low for UCKL-1 and high for MRPS18-2. The samples were cooled at room temperature for 15 min. A two-step IHC procedure was performed with the EnVision FLEX detection system (DAKO), using an automatic staining Link instrument (DAKO). The primary anti-UCKL-1 (diluted 1 : 200) and anti-MRPS18-2 (diluted 1 : 150) antibodies (Sigma-Aldrich) were applied for 60 and 30 min, respectively. The secondary antibody FLEX/HRP (DAKO) was applied for 20 min. The peroxidase enzyme was then visualized with 3,3′-diaminobenzidine, tetrahydrochloride, and hydrogen peroxide. Hematoxylin was used as a counterstain for 10 min. Visual evaluation of the UCKL-1 and MRPS18-2 signals was performed by an experienced pathologist. Each spot was graded individually. The UCKL-1 and MRPS18-2 cytoplasmic reactions were considered as negative if no positive cells were observed. If a MRS18-2 signal was detected mainly in the perinuclear cytoplasm, this was noted in the table of results, as well. The results of IHC reactions were evaluated semiquantitatively, by counting the number of positively stained cells in 1,000 analysed cells as a specified percentage – a label index (LI%). LI values lower than the median expression of the marker were considered as low, and if a LI value overran the median expression it was considered as high.

In addition, demographic information (age, body mass index (BMI), gender), laboratory data on the HCV infection (HCV genotype (GT)), the cumulative size of HCC assessed radiologically, the HCC treatment modality (liver transplantation, liver resection or RFITT), and the time period of follow-up were evaluated for each patient.

**Statistics**

A statistical analysis was performed using the IBM SPSS 19.0 statistical program. The Kolmogorov– Smirnov test was used to assess data normality. Group differences were determined using the Student *t *test when data distribution was normal: in other cases, the Mann Whitney and the Kruskal–Wallis criteria were used. χ^2^ tests were conducted for the categorical variables. To establish a connection between categorical variables, the Spearman correlation coefficient was calculated. A logistic regression model was constructed in order to investigate the association between the intensity of the UCKL-1 and MRPS18-2 signals in the HCC and microvascular invasion, the HCC treatment modality, and HCC recurrence after treatment. All hypotheses were verified with a selected significance level of *p* < 0.05.

## RESULTS AND DISCUSSION

**Characterization of patients**

**Table 1:** Demographic, clinical, and laboratory characteristics of patient groups

Characteristics	HCC (n = 42)	Non-HCC (n = 11)	p-value
Follow up, mean ± SD*, years	2.82±1.76	5.27 ± 2.49	< 0.005
Age, mean ± SD, years	60.1 ± 9.29	49.42 ± 9.29	0.001
Gender, count (rate, %): Women/ Men	16 (38.1%)/ 26 (61.9%)	4 (36.4%)/ 7 (63.6%)	0.917
BMI, mean ± SD, kg/m2	26.32 ± 4.61	26.75 ± 4.18	0.774
HCV genotype (GT*): GT-1, count (rate, %) GT-2, count (rate, %) GT-3, count (rate, %)	27 (64.3) 3 (7.1) 12 (28.6)	9 (81.8) 0 (0) 2 (18.2)	0.314

^*^SD – standard deviation.

Forty-two out of the 53 patients had developed the HCV-associated HCC and received treatment; 11 patients were diagnosed only with HCV-induced cirrhosis. All participants were followed up for at least 1 year at the Santariškiu Klinikos of Vilnius University Hospital; the HCC patients were observed for about 2.82 ± 1.76 years; and the patients with HCV-induced cirrhosis – for 5.27 ± 2.49 years. Noteworthy, the HCC patients were significantly older than the individuals with cirrhosis (*p *= 0.001): the mean age of the HCC patients was 60.1 ± 9.29 years, while the persons in the cirrhosis group were about 49.42 ± 9.29 years old. No differences in gender distribution (male/female ratio) in HCC and non-HCC (cirrhosis) groups were detected (*p *= 0.917). The BMI value was also similar in these groups (*p *= 0.774) (see *Table 1*).

**Table 2:** Histological and immunohistochemical differences in liver tissue samples in groups

Characteristics	HCC (n = 42)	Non-HCC (n = 11)	p-value
HAI, mean ± SD	6.71 ± 1.49	6.64 ± 2.34	0.892
UCKL-1, mean ± SD, LI (%)	50 ± 49.44	24.27 ± 14.53	0.014
MRPS18-2, mean ± SD, LI (%)	8.68 ± 16.61	15.00 ± 15.17	0.260

All participants had histologically confirmed advanced fibrosis, and HAI did not differ between the HCC and non-HCC groups (*p* = 0.892) (*Table 2*).

**Fig. 1:**
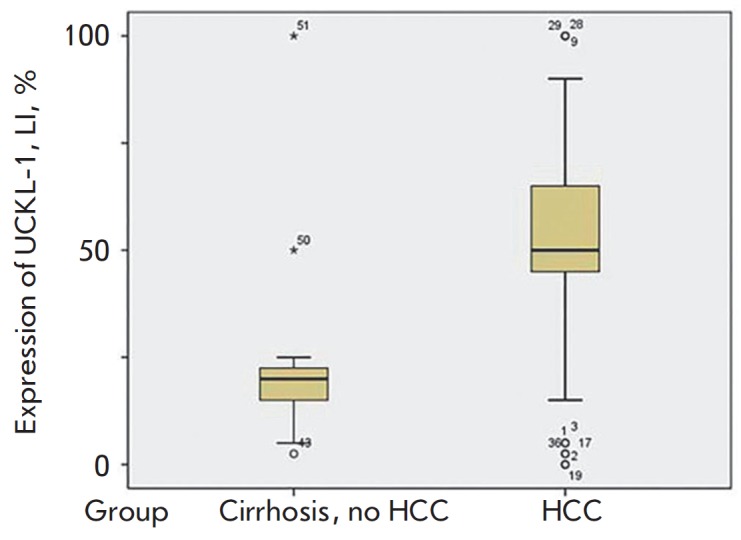
The UCKL-1 expression in the liver tissue. Notice the significant increase in the UCKL-1 staining in samples with HCC in comparison with patients with cirrhosis without HCC

**Fig. 2:**
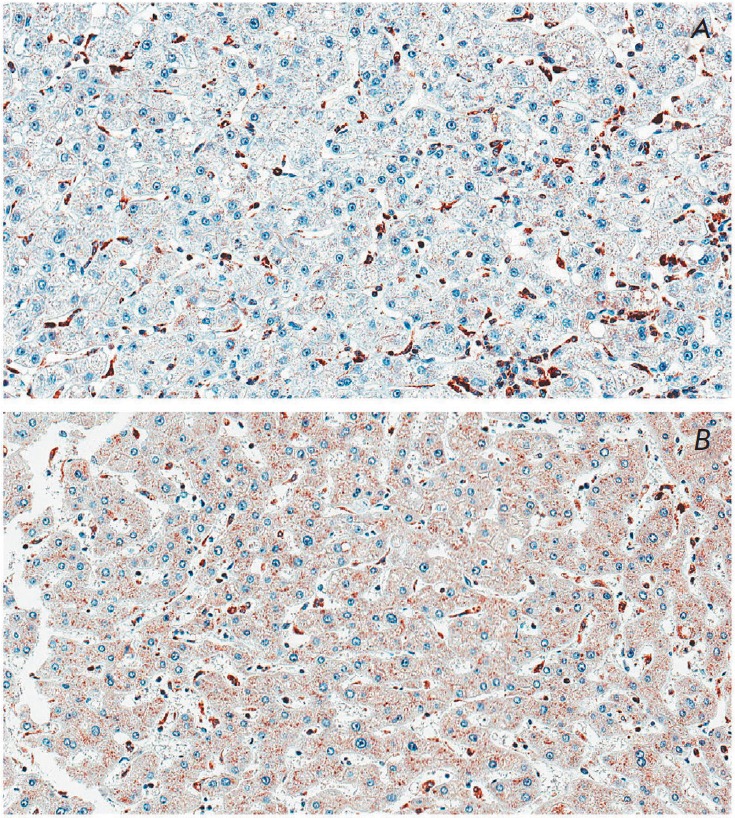
The UCKL-1 cytoplasmic expression in liver tissue. Notice that the UCKL-1 cytoplasmic signal was significantly lower in the hepatocytes (expression was observed in 40% of the cells) of a cirrhosis patient (A) in comparison with the UCKL-1 signal in 100% of the peri-tumor hepatocytes of a HCC patient (B). Objective ×40

The expression of UCKL-1 was high in the liver tissues of patients with HCC. We found that the UCKL-1 signal was stronger in the peri-tumoral liver tissue in HCC cases, compared with non-HCC (cirrhosis) cases (50 ± 49.44 vs. 24.27 ± 14.53, *p *= 0.014), as is presented in *Table 2* and *Fig. 1* and *Fig. 2*.

On the contrary, the MRPS18-2 signal was observed rarely in the liver tissue and this low expression did not differ between the groups (*p*=0.26) (see *Table 2*).

**A description of the cohort of patients with HCC relapse**

HCC recurrence in patients with HCV-induced cirrhosis after curative treatment (liver transplantation, liver resection or RFITT) was confirmed radiologically in 27 of 42 patients. All patients were observed for at least 1 year before the diagnosis of HCC relapse (the mean observation time was 2.93 ± 2.43) (see *Table 3*). HCC relapse appeared usually after 2.76 ± 1.3 years. The recurrence rate (62%) in the studied cohort and the time of relapse were similar to those reported earlier by other authors [10]. Moreover, the mortality rate in the relapsers was significantly higher than that in non-relapsers (59.3 and 6.7%, respectively, *p* = 0.001) (see *Table 3*).

**Table 3:** Characteristics of patient groups with and without HCC recurrence

Characteristics	HCC recurrence (n = 27)	no HCC recurrence (n = 15)	p-value
Follow up, mean ± SD, years	2.76 ± 1.3	2.93 ± 2.43	0.8
Lethality, count (rate, %)	16 (59.3)	1 (6.7)	0.001
Age, mean ± SD, years	63.78 ± 9.22	53.53 ± 4.07	< 0.001
Gender, count (rate, %): -women/men	14 (51.9%)/13 (48.1%)	2 (13.3%)/13 (86.7%)	0.015
BMI average ± SD, kg/m^2^	27.05 ± 4.88	25.3 ± 4.01	0.245
HCC treatment method: -resection, count (rate, %) -RFITT, count (rate, %) -transplantation, count (rate, %)	20 (74.1) 6 (22.2) 1 (3.7)	6 (40) 1 (6.7) 8 (53.3)	0.001

Importantly, the age of the patients in both groups differed significantly: 63.78 ± 9.22 years in those that showed a relapse and 53.53 ± 4.07 years in non-relapsers (*p * < 0.001) (see *Table 3*).

Noteworthy, women were diagnosed with HCC relapse more often than men: 87.5% (14 out of 16) and 50% (13 out of 26), respectively (*p* = 0.015) (see *Table 3*). However, an absolutely larger number of men were diagnosed with HCC (26 men versus 16 women). It had also been reported earlier that older age and male gender are associated with an increased risk of HCC development in HCV cirrhotic patients [21, 22].

In our studied cohort of patients, no differences in BMI values, HAI, and HCC differentiation were detected between the groups of relapsers and non-relapsers (*Table 3*). We have to mention that, in the studied cohort, there were only two cases of poorly differentiated HCC (grade G3) in the group of relapsers and no G3 cases in the non-relapsers. Probably, this is one reason why in our case the histological differentiation grade was not associated with HCC recurrence, contrary to published data [23].

**Table 4:** Histological differences in liver tissue samples in HCC recurrence and non-HCC recurrence groups

Characteristics	HCC recurrence (n = 27)	no HCC recurrence (n = 15)	p-value
HAI, mean ± SD, count	6.89 ± 1.19	6.4 ± 1.92	0.381
HCC size, mean ± SD, mm	50.44 ± 17.831	41.47 ± 20.757	0.558
HCC grade of differentiation -G1, count (rate, %) -G2, count (rate, %) -G3, count (rate, %)	6 (23.07) 18 (69.23) 2 (7.69)	1 (6.67) 14 (93.33) 0 (0)	0.64
Vascular invasion, count (rate, %)	13 (50)	2 (13.33)	0.02

When microvascular invasion was observed, HCC recurrence was diagnosed significantly more often (*p* = 0.02, *Table 4*). As a rule, tumors were larger in HCC relapsers (50.44 ± 17.83 mm vs. 41.47 ± 20.76 mm), but these differences were not statistically significant (*Table 4*). Thus, in our study the microvascular invasion, proven histologically, was an independent predictive factor of a lower disease-free survival rate. Actually, tumor size and vascular invasion are well-known predictive factors of HCC recurrence [24, 25]. The studied cohort in the present paper was rather small, and that could be the reason why the size of the HCC nodules did not differ significantly between relapsers and non-relapsers, even when such a trend was observed.

**The high expression of UCKL-1 and MRPS18-2 in HCC tissues**

**Fig. 3:**
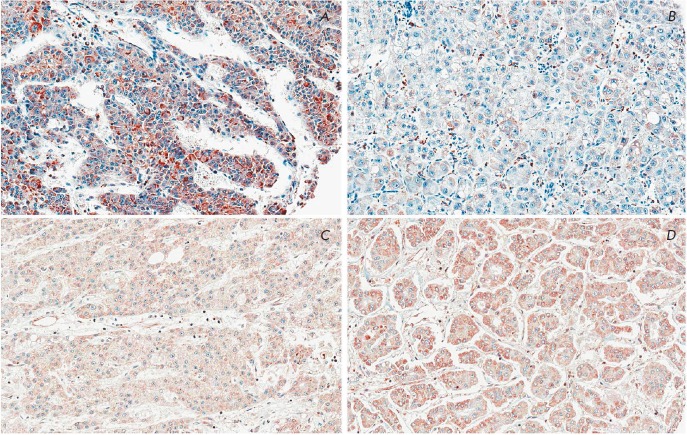
The UCKL-1 and MRPS18-2 expression pattern in cancer tissues. Notice that the UCKL-1 cytoplasmic signal was significantly higher in the HCC samples of relapsers (A) in comparison with the UCKL-1 signal in non-relapsing HCC (B). The MRPS18-2 signal was strong in cancer tissues, regardless relapsing (C) or non-relapsing (D) HCC. Objective ×40

Comparing the expression of UCKL-1 and MRPS18- 2 proteins in HCC nodules, a significantly stronger UCKL-1 signal was observed in HCC relapsers compared with non-relapsers: 62.69 ± 50.4 and 26.0 ± 30.19, respectively (*p *= 0.006). We have to emphasize that, at the same time, in the peri-tumor liver tissue no dramatic differences in UCKL-1 staining were detected when relapsers and non-relapsers were compared (*Fig. 3* and *Table 5*). Hence, the UCKL-1 expression levels might have a prognostic value in terms of HCC occurrence and recurrence.

**Table 5:** Immunohistochemical differences in liver tissue samples in groups of patients with and without HCC recurrence

Characteristics	HCC recurrence (n =27)	no HCC recurrence (n=15)	p-value
UCKL-1, mean ± SD, LI (%)	49 ± 32.44	50.27 ± 14.53	0.510
MRPS18-2 in liver tissue, mean ± SD, LI (%)	9.42 ± 18.239	7.40 ± 13.835	0.583
UCKL-1 in HCC nodule, mean ± SD, LI (%)	62.69 ± 50.4	26 ± 30.19	0.006
MRPS18-2 in HCC nodule, mean ± SD, LI (%)	78.08 ± 54.54	61.67 ± 60.52	0.378

At the same time, the MRPS18-2 expression was several folds greater in the HCC nodules than in the unaffected liver, but no differences were observed between HCC relapsers and non-relapsers (*Table 5*). Therefore, the levels of MRPS18-2 could be considered as lacking prognostic significance for patients with HCV cirrhosis.

**The high expression of UCKL-1 in HCC nodules can be a prognostic factor of HCC relapse**

As was expected based on the data published earlier [26], the method of HCC treatment on its own had a significant predictive value of HCC recurrence: in the case of liver transplantation, the HCC recurrence rate was significantly lower than that after liver resection or RFITT – the were the only cases of HCC recurrence after liver transplantation (*Table 3*). The high rate of tumor recurrence after surgical resection and RFITT corresponded to the data in the literature [27].

**Table 6:** Logistic regression

Characteristics	B	SE	Wald	DV	p-value	Exp(B)
UCKL-1 in HCC	0.029	0.013	5.022	1	0.025	1.029
Vascular microinvasion	2.898	1.176	6.072	1	0.014	18.143
HCC treatment modality (transplantation->resection->RFITT)	2.484	0.933	7.084	1	0.008	11.984
Constant	-6.316	2.178	8.413	1	0.004	0.002

Note. B – regression coefficient, SE – standard error, Wald – Wald statistics value, DV – the dependent variable (1 – for HCC recurrence), Exp (B) – odds ratio.

After a multivariate analysis of prognostic factors for HCC recurrence was performed, we could conclude that the most significant variables were the levels of UCKL-1 expression in tumor nodes, vascular invasion, and the modality of the primary HCC treatment (other than liver transplantation) with odds ratios of 1.029, 18.143, and 11.984, respectively (*R*^2^ = 0.633, *p *= 0.002) (*Table 6*). As has already been mentioned, the expression levels of MRS18-2 and the differentiation of HCC could not be predictive factors for HCC relapse.

Thus, the addition of the expression levels of UCKL- 1 as a predictive factor for the risk of HCC relapse resulted in a better prognosis of the course of the disease. A higher UCKL-1 expression in HCC nodules can be indicative of a higher risk of HCC relapse after curative treatment, especially if the treatment was not liver transplantation.

MRS18-2 was expressed at significantly higher levels in HCC nodules, compared with normal liver tissues, but it was not predictive of HCC recurrence.

These promising results regarding the prognostic value of UCKL-1 in terms of HCC occurrence and recurrence should be confirmed in a larger prospective-retrospective clinical study.

## CONCLUSIONS

A high level of UCKL-1 expression in HCC nodules, in combination with microvascular invasion and HCC treatment modality (other, than liver transplantation), is a predictor of a higher risk of HCC recurrence.
